# Energy expenditure and body temperature variations in llamas living in the High Andes of Peru

**DOI:** 10.1038/s41598-019-40576-9

**Published:** 2019-03-11

**Authors:** Alexander Riek, Anna Stölzl, Rodolfo Marquina Bernedo, Thomas Ruf, Walter Arnold, Catherine Hambly, John R. Speakman, Martina Gerken

**Affiliations:** 1 Institute of Animal Welfare and Animal Husbandry, Friedrich-Loeffler-Institut, Dörnbergstr. 25/27, 29223 Celle, Germany; 2 Department of Animal Sciences, University of Göttingen, Albrecht-Thaer-Weg 3, 37075 Göttingen, Germany; 3 Centro de Estudios y Promoción del Desarrollo del Sur, Calle Malaga Grenet 678 - Umacollo, Arequipa, Peru; 4 Research Institute of Wildlife Ecology, Department of Integrative Biology and Evolution, University of Veterinary Medicine Vienna, Vienna, Austria; 5 Institute of Biological and Environmental Sciences, University of Aberdeen, Aberdeen, AB24 2TZ UK; 6 Institute of Genetics and Developmental Biology, State Key Laboratory of Molecular Developmental Biology, Chinese Academy of Sciences, 100101 Beijing, PR China

## Abstract

Some large herbivores exhibit seasonal adjustments in their energy metabolism. Therefore, our aim was to determine if the llama (one of the most extensively kept livestock breeds) exhibits seasonal adjustment of their energy expenditure, body temperature and locomotion, under its natural high altitude Andean habitat. For this purpose, energy expenditure, body temperature and locomotion were measured in seven non-pregnant llama dams for ten months on the Andean High Plateau (4400 m above sea level). Daily energy expenditure was measured as field metabolic rate using the doubly labelled water method at four different measurement times. Additionally, a telemetry system was used to continuously record activity, body temperature (3 min intervals) as well as the position (hourly) of each animal. The results show that llamas adjusted their body temperature and daily energy expenditure according to environmental conditions. Furthermore, llamas under high altitude Andean climatic conditions exhibited a pronounced daily rhythm in body temperature and activity, with low values at sunrise and increasing values towards sunset. Llamas also had remarkably low energy expenditure compared to other herbivores. Thus, despite the domestication process, llamas have not lost the ability to adjust their body temperature and daily energy expenditure under adverse environmental conditions, similar to some wild herbivores.

## Introduction

Endothermic mammals have to invest a substantial amount of energy to keep their species specific body temperature (T_b_) within a narrow limit of 37–39 °C especially with changing environmental conditions^1^. Therefore, many small mammals in particular those weighing less than ten kilograms, employ energy saving mechanisms such as torpor or hibernation and thus reduce their T_b_ and energy expenditure substantially during harsh environmental conditions^2–5^. Larger animals, with the exception of bears and badgers, were thought not to use such metabolic mechanisms to save energy until some studies on cervid species^6^ and other larger ruminants^7,8^ indicated that they exhibit some form of seasonal adjustment in their metabolism. However, most of these studies were conducted on captive animals using respirometry. In more recent studies, results from free-ranging wild herbivores^9–12^ using telemetry and continuous long-term data recording, suggested that these species are also able to reduce their T_b_ and energy expenditure during unfavorable environmental conditions.

The climate of the Andean Plateau also known as ‘Altiplano’ (altitude >4000 m above sea level, a.s.l.) in South America can be considered as unfavourable to livestock. It is characterised by low annual precipitation of less than 500 mm per year, low ambient temperatures (T_a_) at night falling at times below −20 °C and thus large daily T_a_ amplitudes exceeding 45 °C on some days. Furthermore, vegetation is scarce and low in energy and protein content. The llama (*Lama glama*) and the alpaca (*Vicugna pacos*) are the largest autochthonous herbivores which have been domesticated in South America 6,000–7,000 years ago from their wild ancestors, the guanaco (*Lama guanicoe*) and the vicuña (*Vicugna vicugna*)^13,14^, respectively. Although llamas and alpacas have also been reported to live in lowlands in pre-Columbian times^15^, they are typically concentrated in the high Andean regions. There are currently about 3.3 million llamas living mainly at the Andean High Plateau of Bolivia and Peru^16^ and they are of predominant economic and cultural importance for the rural population^17^. Apart from climatic challenges and feed shortages, llamas and alpacas are also confronted with the impact of high altitude, i.e. reduced atmospheric pressure. Under these conditions, energy efficiency is a prerequisite for survival. In this context, it is noteworthy that South American camelids have been shown to possess an extraordinary high blood oxygen affinity^18^.

Although there exists a large body of scientific literature on South American camelids on health, nutrition and reproduction in temperate regions (for review see Fowler 2010^19^), there is still a large gap in scientific knowledge on how these animals adapt to the harsh environment of the high Andes. Therefore, the aim of our long-term study was to determine if the llama, exhibits seasonal and/or daily adjustment mechanisms with regard to energy expenditure and T_b_ in its natural habitat of the high Andes in South America.

## Results

### Climatic conditions

**Figure 1:**
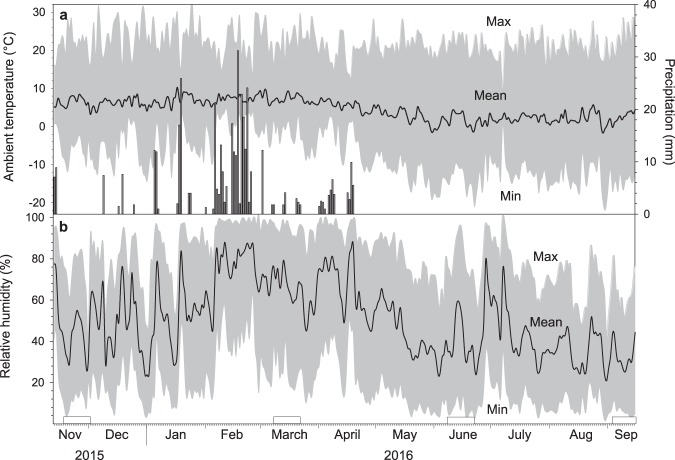
Climatic variables during the study in the Andes of Peru. (**a**) Average daily ambient temperature, (**b**) average daily relative humidity (black lines) with daily maxima and minima (grey shaded area) and precipitation (black bars) during the course of the study (308 days) at the study location in the high Andes of Peru (4400 m a.s.l.). Rectangles denote field metabolic rate measurement periods.

The climatic conditions during the time of our study (13 Nov 2015–15 Sep 2016) were typical for the Andean High plateau with very low T_a_’s during the night and high T_a_’s during the day (Fig. 1a). Average daily T_a_ over the entire study period was 4.6 ± 2.7 °C and ranged from −3.7 °C to 10.3 °C. The mean daily minimum T_a_ during our study was −8.1 ± 6.1 °C and ranged from −22.1 °C to 4.6 °C. During the entire study of 308 days, there were 263 days with frost. Mean daily maximum T_a_ was 22.2 ± 3.6 °C and ranged from 9.6 °C to 32.7 °C. The amplitude of daily T_a_ fluctuations, i.e. the difference between daily maximum and minimum T_a_ during the time of the study averaged 30.1 ± 7.3 °C and ranged from 9.5 °C to 45.2 °C. The mean daily relative humidity (RH) was 50.9 ± 17.6%, mean daily maximum RH was 81.0 ± 14.1% (range 41.8–100.0%) and mean daily minimum RH was 17.7 ± 12.4% (range 0.65–61.4%; Fig. 1b). The total precipitation during our study was 424 mm. Precipitation occurred exclusively during the wet season from November to April on 54 of the 308 study days (Fig. 1a). The highest rainfalls occurred on 18 February (31 mm) and 19 January (25 mm). Rainfall on the remaining days ranged between 1 and 18 mm. Natural daylight during our study ranged from 10 to 12 h per day.

### Rumen temperature

**Figure 2:**
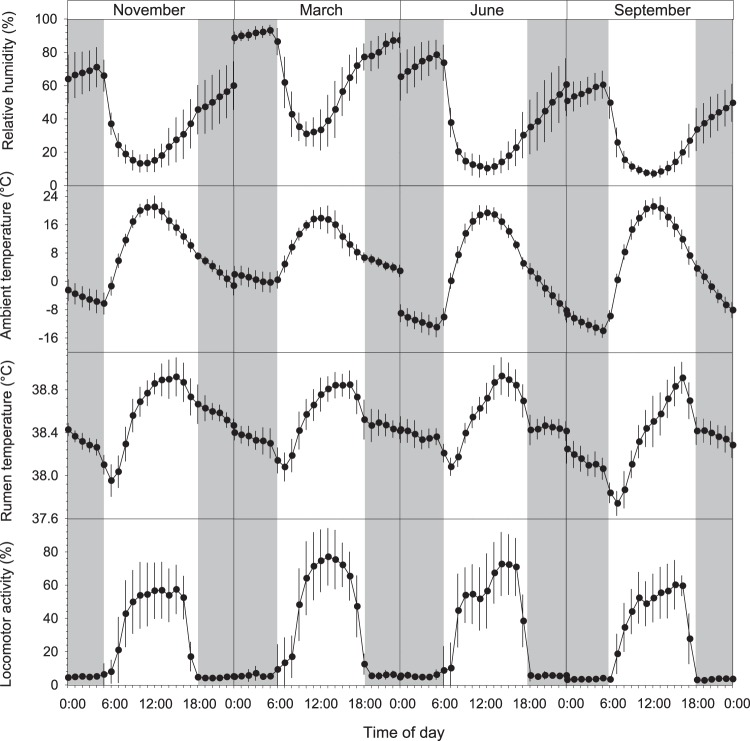
Average diurnal rhythms of relative humidity, ambient temperature, rumen temperature and locomotor activity. Data were collected during the FMR measurements of 15 days each in November, March, June and September in llamas (*n* = 7) under high Andean climatic conditions in Peru (4400 m a.s.l.). Values are hourly means ± *se*. Grey shaded areas indicate night-phase.

**Figure 3:**
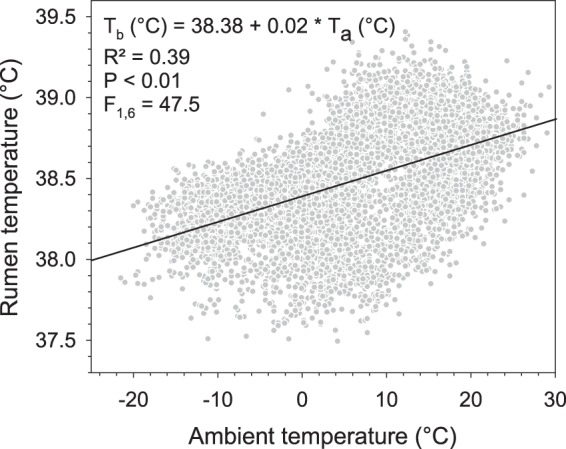
Relationship between rumen temperature and ambient temperature. Data are hourly means from seven adult non-pregnant llama dams (*n* = 51744) under high Andean climatic conditions (rumen temperature was taken at 3 min intervals during 308 days of sampling). Slope and intercept are adjusted for repeated measurements of individual animals (see text for details).

**Figure 4:**
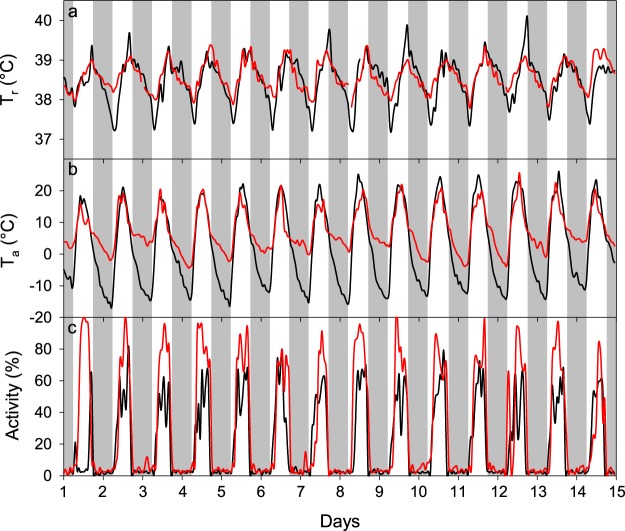
Examples of the diurnal rhythm of the (**a**) mean rumen temperature (T_r_), (**b**) ambient temperature (T_a_) and (**c**) locomotor activity. Data are from adult non-pregnant llama dams (*n* = 7) during the field metabolic rate measurements in March (red line) and September (black line). Grey shaded areas indicate night-phase.

Over the entire study period, we collected over 760,000 rumen temperature (T_r_) measurements at 3 min intervals, ranging from 36.25 °C to 41.17 °C. The average daily T_r_ during our study was 38.46 ± 0.25 °C (Table 1). The T_r_ followed a diurnal rhythm with the lowest T_r_ usually just after sunrise and the highest T_r_ around late afternoon (Fig. 2). Comparing the minimum T_r_ and maximum T_r_ between months, the lowest recorded minimum T_r_ occurred in September (36.25 °C) and the highest maximum T_r_ in June (40.81 °C). The T_r_ amplitude, i.e. the difference between daily maximum T_r_ and daily minimum T_r_, was very variable and reached on some days more than 3 °C, increasing from November to September over the entire study. This trend was also evident during the FMR measurements, i.e. the T_r_ amplitude was significantly (P < 0.001) lower in November and March compared to June and September (Table 1, Fig. 2). There was a significant positive relationship between T_r_ and T_a_ over the entire study period (T_r_, °C = 38.38 + 0.02 * T_a_, °C; R² = 0.39, F_1,6_ = 47.5, P < 0.01, n = 51744, Fig. 3). An example of the adjustment of T_r_ to T_a_ is given in Fig. 4. The figure shows that on days with low T_a_ amplitudes for high Andean conditions as it was the case in March with only 23 °C, T_r_ amplitudes decreased as well even though locomotor activity (LA) was high. Contrarily, on days with large T_a_ fluctuations of more than 37 °C such as in September during the dry season, T_r_ decreased at night much further compared to March.

**Table 1:** Rumen temperatures in llamas in the high Andes of Peru.

Variable	Entire study (308 days)	Periods of FMR measurements (15 days each)				*SEM*	*F*_*(3, 17)*_-value	Months *p*-value
		November	March	June	September			
Average daily T_r_	38.46 ± 0.25	38.47^a^	38.49^a^	38.49^a^	38.31^b^	0.06	4.51	0.018
Range	37.79–40.11	38.27–38.98	38.04–38.77	38.05–39.02	37.85–38.95			
Average daily T_r_ minimum	37.75 ± 0.35	37.83^a^	37.86^a^	37.83^a^	37.54^b^	0.08	3.72	0.030
Range	36.25–39.00	37.06–38.44	36.75–38.19	36.94–38.62	36.88–38.31			
Average daily T_r_ maximum	39.19 ± 0.34	39.05	39.12	39.24	39.13	0.09	2.05	0.147
Range	38.49–41.17	38.69–39.88	38.68–39.94	38.81–41.17	38.62–40.12			
Average daily T_r_ amplitude	1.44 ± 0.41	1.23^c^	1.27^c^	1.40^b^	1.61^a^	0.10	7.96	<0.001
Range	0.62–3.44	0.75–2.50	0.63–2.44	0.75–3.22	0.83–3.06			

Rumen temperature (T_r_, °C) variables in llama dams (*n* = 7) during the entire study of 308 days and during the field metabolic rate measurements of 15 days each in November, March, June and September under Andean climatic conditions in Peru. Values are means ± *sd* for the entire study and LS-Means for the months adjusted for repeated measurements of seven animals with the corresponding *SEM*, *F*- and *p*-value.

^a,b,c^Means within a row between month not sharing the same superscript differ by *P* < 0.05.

### Field metabolic rate and water turnover

**Figure 5:**
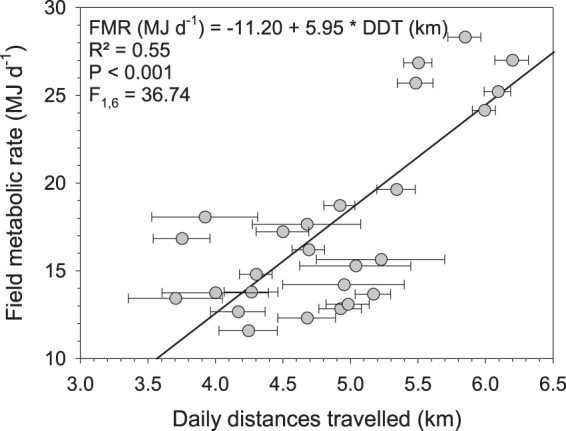
Relationship between field metabolic rate (FMR) and daily distances travelled (DDT). Data are means ± *se* from adult non-pregnant llama dams at four different measurement periods of 15 days each under high Andean climatic conditions (4400 m a.s.l.; *n* = 26; 6 animals in November, 7 in March, 6 in June and 7 in September). Slope and intercept are adjusted for repeated measurements of individual animals (see text for details).

The field metabolic rate (FMR) varied between the four different measurement periods of 15 days each (Table 2). The lowest and highest individually recorded FMR were 11.6 MJ d^−1^ and 28.3 MJ d^−1^, respectively. In June, during the dry season, when average T_a_ amplitudes were high (35.35 ± 2.67 °C) and animals traveled on average longer daily distances (5.83 ± 0.28 km), FMR was significantly higher (26.22 ± 1.48 MJ d^−1^) compared to the measurements during the wet season, i.e. November (13.15 ± 1.77 MJ d^−1^) and March (15.43 ± 1.84 MJ d^−1^). The FMR values measured during the wet season in November and March did not differ (P = 0.13), however they did differ (P < 0.001) between the two measurements during the dry season (i.e. June and September). In general, FMR values were higher during the dry than during the wet season (Table 2). Mixed model analysis revealed that daily distances travelled (P < 0.001, F_1,6_ = 36.74, Fig. 5), average T_a_ (P < 0.01, F_1,6_ = 17.44), average minimum T_a_ (P < 0.05, F_1,6_ = 7.81) and average maximum T_a_ (P < 0.01, F_1,6_ = 15.46) had significant effects on FMR.

**Table 2:** Average physiological and behavioural variables in llamas in the high Andes of Peru.

Variable		November	March	June	September	*SEM*	*F*_*(3, 17)*_-value	Months *p*-value
Body mass	(kg)	125.4^a^	109.9^b^	117.2^ab^	125.5^a^	5.16	5.62	0.007
Body condition score	(points)	2.48	2.27	2.46	2.66	0.18	1.74	0.195
Field metabolic rate	(MJ d^−1^)	13.15^c^	15.43^bc^	26.22^a^	16.19^b^	0.79	61.91	<0.001
Total body water	(%)	61.44^b^	66.75^a^	65.85^ab^	64.45^ab^	1.52	3.40	0.041
Total water intake	(L d^−1^)	4.65^a^	5.00^a^	5.20^a^	3.75^b^	0.20	19.23	<0.001
Daily activity 24 h	(%)	25.05^b^	29.71^a^	28.07^a^	23.05^b^	0.80	26.33	<0.001
Day	(%)	43.69^b^	53.10^a^	50.26^a^	42.91^b^	1.92	20.15	<0.001
Night	(%)	4.82^b^	6.30^a^	5.53^a^	3.56^b^	0.11	30.24	<0.001
Distance travelled^1^	(km d^−1^)	4.34^b^	4.50^b^	5.83^a^	4.84^b^	0.17	23.24	<0.001
Daily rumen temperature	(°C)	38.47^a^	38.49^a^	38.49^a^	38.31^b^	0.06	4.51	0.018
Daily ambient temperature	(°C)	6.71 ± 1.21	7.46 ± 2.85	1.99 ± 1.79	5.28 ± 1.30			
Daily minimum temperature	(°C)	−6.74 ± 2.87	−1.20 ± 2.22	−13.52 ± 2.52	−14.46 ± 1.71			
Daily maximum temperature	(°C)	23.30 ± 2.72	21.96 ± 2.85	21.83 ± 2.32	23.16 ± 3.37			
Daily temperature amplitude	(°C)	29.93 ± 4.58	23.17 ± 4.12	35.35 ± 2.67	37.62 ± 3.87			

Data are averages from seven llama dams during 15 days in four different months under Andean climatic conditions in Peru. Values are LS-Means with the corresponding *SEM*, *F*- and *p*-value. Additionally average ambient temperature variables for the respective time periods are given (means ± *sd*).

^1^For November and June averages are from six animals.

^a,b,c^Means within a row not sharing the same superscript differ by *P* < 0.05.

Total body water of individual animals ranged from 56 to 71% of body mass. Average total body water was significantly lower in November (61.4 ± 5.35%) compared to March (66.8 ± 4.0%), but did not differ between all other measurement months (Table 2). Similarly, total water intake (TWI) in September was 3.75 ± 0.51 L d^−1^, significantly lower compared to all other measurement months, while TWI did not differ between November, March and June.

### Locomotor activity and distances covered

In our study, animals were herded to (07.00 h) and from (17.00 h) the grazing grounds every day approximately at the same time, thus LA followed a strong diurnal pattern over the entire study period, similar to T_a_. An example of that pattern for LA as well as for T_a_ and T_r_ is depicted in Fig. 4. During the FMR measurements average daily LA was significantly higher (P < 0.001) during March (29.71 ± 1.80%) and June (28.07 ± 1.38%), compared to November (25.05 ± 1.59%) and September (23.05 ± 2.75%). The same trend was evident when dividing the data into night (i.e. when animals stayed in the corral) and day (i.e. when animals were out grazing; Table 2).

Average daily distances traveled by the animals varied over the study period. Over the entire study the mean daily distance travelled was 4.67 ± 1.41 km and ranged from 1.03 km to 12.06 km. During the FMR measurements the daily distances travelled in June were significantly (P < 0.001) higher compared to all other FMR measurement months, but no differences (P > 0.05) were detected between November, March and September (Table 2).

## Discussion

Our study is the first measuring FMR using the doubly labelled water method in the llama in its natural habitat of the Peruvian high Andes. Furthermore, we combined FMR data with data from a telemetry system measuring T_r_, activity and distances traveled by GPS. These are the first continuously recorded long-term T_r_ and activity measurements for a camelid in the high Andes. Our data show that llamas spend substantially more energy when traveling long distances. However, compared with other ruminants and herbivores llamas have a lower FMR. Furthermore, considering the harsh climate of the Andes, llamas seem to adjust their T_b_ according to T_a_ to save energy.

Our present data on FMR in llamas kept in their natural habitat of the Andes are similar to results reported recently for llamas measured in a temperate lowland environment^20^, which ranged from 17.48 to 25.87 MJ d^−1^. However, considering the much larger daily T_a_ fluctuations in the high Andes (Fig. 1), the present FMR values suggest that llamas adjusted their FMR according to T_a_. Several studies have reported reductions in FMR in domestic and wild ungulates during adverse environmental conditions^10–12,21^. Our results from llamas in the Andes support these findings. The range of T_a_ in which T_b_ is regulated by sensible heat loss and thus does not require additional energy for thermoregulation is called the thermal neutral zone (TNZ). Although the TNZ of the llama has not been measured, results from guanacos, which is the wild ancestor of the llama, suggest that the TNZ lies somewhere in the range of −15.5 to 20 °C^22,23^, i.e. −15.5 °C being the lower critical temperature and 20 °C the upper critical temperature outside which the animal needs additional energy to regulate T_b_. Assuming a similar TNZ for the llama, animals in our study were outside their TNZ for some portions of the day during all FMR measurement periods when average T_a_ increased above 20 °C (Table 2). Thus, the increased FMR measured in June and September can be partially explained by the increased average T_a_ amplitudes as evidenced by correlations between the FMR and T_a_ variables. However, it needs to be emphasised that these are average T_a_ variables over FMR measurement periods of 15 days each. On some individual days during the FMR measurements T_a_ ranged between −19 °C and 28 °C and thus were even further outside the suggested TNZ. The FMR measured in June (26.22 ± 1.48 MJ d^−1^) was nearly 100% higher than that in November (13.15 ± 1.77 MJ d^−1^). This can partly be explained by the longer distances the animals travelled in June compared to all other measurement periods (Table 2, Fig. 5). However, FMR was significantly affected by T_a_ and thus animals seemed to have increased their energy expenditure not only due to the longer distances traveled but also due to differences in T_a_.

**Figure 6:**
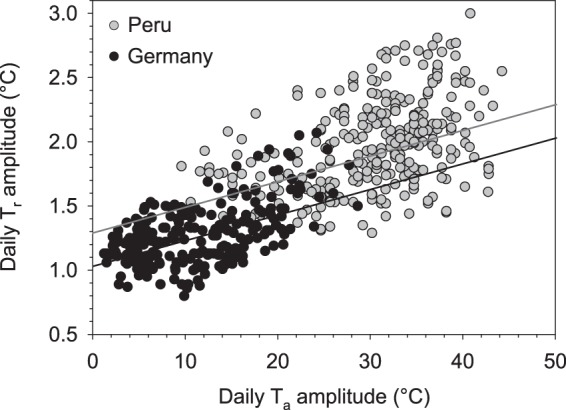
Comparison of temperature amplitudes in llamas between two study locations. Relationship between daily rumen temperature (T_r_) and daily ambient temperature (T_a_) amplitudes at the two different study locations in Germany (black dots, black line: Daily T_r_ amplitude = 1.03 + 0.02 * daily T_a_ amplitude, R² = 0.25, F_1,6_ = 12.84, P < 0.01) and Peru (grey dots, grey line: Daily T_r_ amplitude = 1.29 + 0.02 * daily T_a_ amplitude, R² = 0.22, F_1,6_ = 10.79, P < 0.05). Data are means of seven animals and the respective T_a_ amplitude of that day (Germany, 365 days; Peru, 308 days). Slopes and intercepts are adjusted for repeated measurements of individual animals (see text for details).

The course of daily T_a_ in our study was typical for the High Andean climate with very low T_a_ at night and moderate to high T_a_ during the day (Figs 1 and 2). Thus daily T_a_ amplitudes reached 45 °C on some days. With increasing T_a_ amplitudes, T_r_ amplitudes increased as well, similar to results found in a previous study on llamas kept in a temperate environment^20^. However, the daily T_a_ and T_r_ fluctuations in the previous study were much smaller compared to the present results. Although a comparison between both locations has to be treated with caution (due to random effects etc.), the data show that T_r_ and T_a_ amplitudes were correlated in both studies (Fig. 6). The results from the high Andes, however, suggest a higher flexibility in regulating T_r_ according to T_a_ in llamas kept at these altitudes (~4400 m a.s.l.).

In our study T_r_ decreased during the night and increased during the day. These daily T_r_ fluctuations were higher during the dry season (May–September) than during the wet season (November–April) and similar to the T_a_ fluctuations (Figs 2 and 4), suggesting that animals followed a shallow daily hypometabolism. Reducing the metabolic rate to save energy has been known for a long time to be employed by many small mammals weighing less than 10 kg (for review see Heldmaier *et al*.^3^; Ruf and Geiser^5^, Geiser^24^) but not for larger animals with the exception of bears and badgers. But there is increasing evidence, that also larger mammals such as red deer^10^, ibex^11^ and horses can reduce their metabolic rate to save energy. The average daily T_r_ fluctuations we report here were lowest in November (1.44 °C) and highest in September (1.61 °C, Table 2). These values are in the range of previously reported T_b_ amplitudes for zebras (1.7 °C)^25^ alpacas (1.5 °C)^26^, angora goats (1.4 °C)^27^, blesbok (1.4 °C)^26^, impalas (1.1 °C)^28^ and pronghorn (1.0 °C)^29^. However, these values and our results are means of several animals over a number of days. The highest individual T_r_ amplitudes in our study over a period of ten months ranged from 2.50–3.44 °C (Table 1). Even higher amplitudes of 4–7 °C have been found for the Arabian oryx^8^, springbok^30^ and camel^31^. The daily T_r_ fluctuations observed in our study were larger than the circadian variations of llamas under temperate conditions (37.5–38.6 °C)^32^ and suggest that the animals used heterothermy, possibly to reduce energy expenditure. Furthermore, these daily T_r_ fluctuations followed the daily photoperiod and daily T_a_ cycle over the entire study period as evidenced by the correlation between T_r_ and T_a_ (Fig. 3). Similar results have been also found for ibex^11^, red deer^10^ and horses^9,33–35^. Because animals were herded every morning at around the same time to the pastures, activity increased sharply at that time and thus possibly resulted in an increase of T_r_. In earlier studies on herbivores, T_b_ or T_r_ fluctuations decreased with decreasing average T_a_ ^10,11^. In the present study, however, daily T_r_ fluctuations increased with decreasing average T_a_ and higher T_a_ amplitudes (Tables 1 and 2, Figs 2 and 4). The increased T_r_ amplitudes could be explained by a decrease in pasture quality during the dry season. Thus, energy needs might have been compromised, which could have led to increased heterothermy by lowering the minimum T_r_ and thus increasing the T_r_ amplitude^26^. However, our body mass and body condition score data do not support this suggestion (Table 2). Therefore, it is more likely that animals lowered their T_r_ at night to increase the capacity to store heat during the day and thus reducing energetic costs as has been shown in a number of herbivores such as the eland^36^, Arabian oryx^37^, giraffe^38^, Arabian sand gazelle^39^, Thompson’s gazelle, Grant’s gazelle^40^ and the Asian elephant^41^.

**Figure 7:**
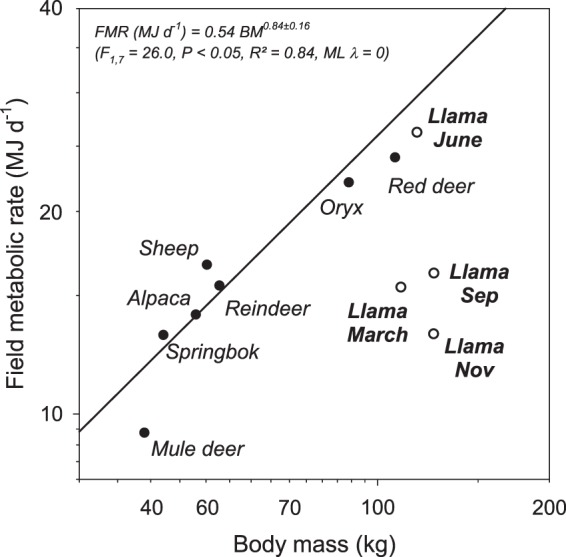
Relationship between field metabolic rate (FMR, measured using the doubly labelled water method) and body mass in herbivores. Data are from seven herbivores (black dots) published elsewhere (see text for details) and from the llama of the present study (black circles) at four different measurement periods under high Andean climatic conditions (4400 m a.s.l.). The regression line was derived using the phylogenetic least square approach, excluding the data from the llama. However, the maximum likelihood (ML) of lambda was 0 and thus the regression line represents an ordinary least square regression.

In an earlier study^20^ llama FMR measured in a temperate European environment was compared with the FMR of other herbivores published so far measured using the doubly labelled water method under natural conditions (Mule deer, *Odocoileus hemionus*^42^; reindeer, *Rangifer tarandus*^43^; springbok, *Antidorcas marsupialis*^44^; red deer, *Cervus elaphus*^45^; Arabian oryx, *Oryx leucoryx*^8^; sheep, *Ovis aries*^46^; alpacas, *Lama pacos*^47^). Based on these data, a phylogenetic corrected regression equation was derived (FMR, MJ d^−1^ = 1.23 BM^0.63±0.12^) from which a predicted FMR of 28.9 MJ d^−1^ for the llama could be computed. The predicted FMR was about 10% and 30% higher than the actual measured FMR in that study in summer and winter, respectively. In the present study, we derived a separate phylogenetic corrected regression equation (Fig. 7). The resulting regression line predicted FMR values for llamas of 31.34 MJ d^−1^, 28.05 MJ d^−1^ and 31.37 MJ d^−1^ for November, March and September, respectively. These predicted values were 138%, 81% and 93% higher compared to the actual measured FMR values for November, March and September, respectively. The measured FMR in June (the highest of the four measurements) however was with 26.22 MJ d^−1^ just 11% lower compared to the predicted FMR from the regression line (29.53 MJ d^−1^). Thus, the three measurements from November, March and September were exceptionally low, compared to values from other herbivores, with the exception of the mule deer. As already suggested in previous studies^48^, camelids in general and the llama in particular seem to have exceptionally low energy expenditures compared to other herbivores, which might be an adjustment to the harsh Andean climatic conditions and low food supply at high altitudes. An even lower FMR has been reported for the giant panda^49^. Contrarily, predicted FMR values from phylogenetic corrected regression equations for alpacas did not deviate much from actual values (Fig. 7). The relative higher FMR in alpacas compared to llamas might be due to their additional metabolic requirements for fine fibre production. In this context studies on high altitude adaptation of oxygen transport properties of blood and circulation could give further insight into the energy metabolism in camelids. Among other features, a high blood oxygen affinity assures a sufficient blood saturation. Interestingly, many of the special blood and circulation properties found in South American camelids are also described for camels^18,19^. However, camels do not live in high altitudes, but Old and New world camelids share their capability to survive in arid climates.

The TWI calculated during the FMR measurement periods did not differ between November, March and June but was significantly lower in September, i.e. at the end of the dry season (Table 2). Interestingly T_r_ amplitudes were highest at the end of the dry season in September and FMR decreased during the dry season from June to September (Table 2), suggesting that animals not only conserved energy but also water towards the end of the dry season. This is in agreement with previous studies suggesting that animals, especially camelid species with a pronounced low metabolism living in resource poor environments have an adaptive advantage because not only less energy resources are required but also less water is lost during respiration^48^.

In conclusion, our study provides evidence that llamas kept at the Andean High Plateau have an exceptionally low energy expenditure compared to other ruminants. Furthermore, llamas seem to adjust their T_b_ according to T_a_ which must involve some trade-offs that allow them to save energy instead of keeping their T_b_ constant. Understanding these trade-offs may provide further insights into the adaptations of animals allowing them to survive in extreme environments such as the high Andes.

## Methods

### Animals and study site

Procedures performed in our study were in accordance with the Peruvian animal ethics regulations and approved by the Peruvian National Ministry for Health (SENASA 2016-0009809). The study was conducted for 308 days from November 2015 to September 2016 at the research station Toccra (Centro de Desarrollo Alpaquero de Toccra) of the non-governmental organisation DESCOSUR (Centro de Estudios y Promoción del Desarollo del Sur, Arequipa, Peru) at an altitude of 4400 m a.s.l., approx. 80 km to the North of the city of Arequipa in the Andes of Southern Peru (15°44′21″S, 71°26′33″W). The study area is characterised by a semi-arid climate with an average annual rainfall of 400–500 mm and T_a_ ranging from as low as −25 °C at night to as high as 30 °C during day time. The average year is divided into a wet season (November–April) when nearly all of the annual rainfall occurs and a dry season (May–October).

Study animals originated from a large female llama herd of 210 animals kept under a traditional Andean herding system, i.e. animals were led to pasture in the morning shortly after sunrise at approx. 07:00 h and were herded back into a corral before sunset at approx. 17:00 h where they stayed throughout the night partly to protect them from their only predator, the nocturnal puma (*Puma concolor*). During the day animals roamed freely on the pasture of the High Andean plateau consisting mainly of the ecosystems *pajonal* (dry with tall bunch grasses) and *bofedal* (wet with grasses and herbs). The *bofedales* are formed by impenetrable stone and clay layers upon which melting water accumulates. No additional feeding was practiced and water was available throughout the year by natural surface water. For the present study a total of seven non-pregnant adult llama dams with an average age of 5.7 ± 1.5 years and a mean body mass of 125.4 ± 15.2 kg were randomly chosen and kept together with the rest of the herd.

### Measurements

#### Climate

The T_a_ (resolution: 0.0625 °C) and RH (resolution: 0.04%) were recorded continuously throughout the study with miniature data loggers at 30 min intervals at approx. 1.5 m above the ground (i-Button, DS1923#F5, Maxim Integrated Products, Sunnyvale, CA, USA). Precipitation data were obtained from a nearby weather station at approx. 10 km distance to the farm (15° 58′43″S, 71° 12′48″W).

#### Telemetry and body condition score

We equipped seven animals with a telemetry system (GPS Plus-3 Store on Board collar, Vectronic Aerospace GmbH, Berlin, Germany) described in detail elsewhere^50^. In brief, the telemetry system consists of two units, a ruminal unit (22 × 80 mm, 100 g) and a collar unit (450 g). The ruminal unit was administered *perorally* after animals were immobilized with an anaesthetic drug (Xylacin, Rompun®; Bayer HealthCare, Leverkusen, Germany, 4 mg/100 kg body mass). The ruminal unit measured T_r_ every 3 min, which was transmitted via short-distance UHF link to a data logging system located in the collar unit^50^. Furthermore, LA was also recorded every 3 min with two different activity sensors and expressed in % of the maximum value recorded. All data were recorded for 308 days and stored in the collar unit and downloaded via a laptop. Additionally the position of each animal was recorded every 60 min using a GPS device located on the back of the collar (GPS Plus-3 Store on Board collar, Vectronic Aerospace GmbH, Berlin, Germany). The body condition score, a palpable and visual assessment of the degree of fatness of individual animals was recorded during the four FMR measurement times according to a point system (scale: 0 = emaciated to 5 = obese) described in detail elsewhere^51^.

#### Field metabolic rate

The FMR, total body water and TWI were determined during 15 days at four different time periods during the study i.e. 17 November–1 December 2015, 7–21 March 2016, 7–21 June 2016 and 2–15 September 2016, for each animal using the doubly labelled water (DLW) method^52,53^. At the beginning and at the end of the FMR measurements, body mass was recorded for each llama using a mobile scale (Weighing System MP 800, resolution: 0.1 kg, Patura KG, Laudenbach, Germany) and a blood sample of 5 ml was drawn from the *Vena jugularis* of every animal to estimate the background isotopic enrichment of ^2^H and ^18^O in the body fluids (method D; Speakman and Racey^54^). After taking the background sample, each llama was injected intravenously with approximately 0.16 g of DLW per kg body mass, (65% ^18^O and 35% ^2^H). The individual dose of each llama was determined prior to the injection according to its body mass. The actual dose given was gravimetrically measured by weighing the syringe before and after administration to the nearest 0.01 g (Digital Scale LS200, G&G GmbH, Neuss, Germany). The llamas were then held in a corral with no access to food or water for an 8-h equilibration period, after which a further 5 ml blood sample was taken. After dosing, additional blood samples were taken at 7 and 15 days to estimate the isotope elimination rates.

All blood samples were drawn into EDTA blood tubes. Whole blood samples were transported to the city of Arequipa and were pipetted into 1 ml glass vials and stored at −20 °C until determination of ^18^O and ^2^H enrichment. Samples were sent from Peru to Europe by airmail. Blood samples were vacuum distilled^55^, and water from the resulting distillate was analysed using a Liquid Isotope Water Analyser (Los Gatos Research, USA) at the University of Aberdeen, Aberdeen, Scotland, UK. Samples were run alongside five lab standards for each isotope and IAEA International standards (SMOW, GISP and SLAP) to correct for daily machine variations and delta values were converted to ppm. Isotope enrichments were converted to values of CO_2_ production using a two pool model as recommended for this size of animal^56^. We chose the assumption of a fixed evaporation of 25% of the water flux, since this has been shown to minimize error in a range of applications^57,58^. Specifically carbon dioxide production rate (r_CO2_) per day in mols was calculated using equation A6 from Schoeller *et al*.^59^. The daily amount of energy expended measured as FMR was calculated from carbon dioxide production by assuming a respiration quotient of 0.85. Total body water (mols) was calculated using the intercept method^53^ from the dilution spaces of both oxygen and hydrogen under the assumption that the hydrogen space overestimates total body water by 4% and the oxygen-18 space overestimates it by 1%^59^. The TWI (l/day) that consists of drinking water, preformed water ingested in food and metabolic water was estimated as the product of the deuterium space and the deuterium turnover rate^60^.

### Statistical Analysis

The measurements of T_r_ had declines that could be attributed to the ingestions of water and cold food. These data points were removed by visually checking the raw data. In this cleaned data set, T_r_ values ranged from 36.25 to 41.17 °C. In total 2156 individual days were available for data analysis of LA and T_r_. For each animal, hourly and daily means were calculated using R 3.5.0^61^.

To compare T_r_ (Table 1) and various physiological and behavioural variables (Table 2) during the time of FMR measurements a mixed model was used with animal as a random factor to adjust for repeated measurements and month (i.e. FMR measurement periods) as a fixed factor using the MIXED procedure in SAS version 9.4 (SAS, Inst. Inc., Cary, NC). An integrated post-hoc test (Tukey) was used to detect differences between means with a 5% significance level. Data are expressed as LS-Means or means ± sd where appropriate. To adjust for repeated measurements in all other analysis we included animal ID as a random factor in a mixed model using the MIXED procedure in SAS. Thus, slopes and intercepts in Figs 3, 5 and 6 are adjusted for repeated measurements. Additionally we included body mass as a covariate and month as a fixed factor in a mixed model analysis to test whether various variables had an effect on FMR. Daily distances between continuous GPS locations for each animal were calculated with the program package ‘geosphere’^62^ in R version 3.5.0^61^.

To test for the generality of the relation between body mass and FMR in herbivores, published data and our results were assessed using the PGLS approach in order to account for the potential lack of independence between species, because of their shared evolutionary history. The statistical procedure has been described in detail elsewhere^63–67^. In brief, the phylogeny was derived from a published mammalian supertree which includes 4510 species with updated branch lengths derived from dated estimates of divergence times^68^. The supertree for mammals was pruned to include only the species of concern, i.e. herbivores (*n* = 8), using the ‘Analysis in phylogenetics and evolution’ package (APE^69^) and the ‘Analysis of evolutionary diversification’ package (GEIGER^70^) in R. The method of PGLS was implemented for the trait data using the ‘Comparative analyses of phylogenetics and evolution’ package (CAPER^71^) in R using Pagel’s branch length transformations (lambda, λ)^72^.

## Data Availability

The data analysed during the current study are available from the corresponding author on reasonable request.
